# Causal relationship between depression and metabolic dysfunction-associated steatotic liver disease: a bidirectional Mendelian randomized study

**DOI:** 10.3389/fpsyt.2024.1384003

**Published:** 2024-06-06

**Authors:** Weiyu Liang, Kunting Zhong, Tingting Lai, Yuhao Zeng, Zhanhui Huang, Jiqing Zhou, Jin Huang, Zhenni Shi, Jin Zhang, Fuping Ding

**Affiliations:** ^1^ Research Centre of Basic Integrative Medicine, School of Basic Medical Sciences, Guangzhou University of Chinese Medicine, Guangzhou, China; ^2^ Traditional Chinese Medicine Department, Yihui Fund Hospital, Shanwei, China; ^3^ Clinic Department of Guangzhou University of Chinese Medicine, Guangzhou, China; ^4^ School of Nursing, Guangzhou University of Chinese Medicine, Guangzhou, China

**Keywords:** two-sample Mendelian randomization, depression, nonalcoholic fatty liver disease, metabolic dysfunction-associated steatotic liver disease, liver enzyme

## Abstract

**Background:**

With the global rise in obesity, metabolic dysfunction-associated steatotic liver disease (MASLD) has emerged as the most common chronic liver disease. Concurrently, depression is a highly prevalent mental disorder. As the incidence of MASLD and depression continues to increase, a growing body of research indicates a potential association between the two conditions. However, the direction of causality between depression and MASLD remains uncertain. To address this gap, our study utilizes a two-sample Mendelian randomization (MR) approach to explore the bidirectional causal relationship between depression and MASLD.

**Methods:**

We extracted single nucleotide polymorphisms (SNPs) associated with depression and MASLD from pooled data of genome-wide association studies (GWAS). A comprehensive assessment of possible causality was also performed. Possible mediating effects of liver enzymes on MASLD were also assessed.

**Results:**

A total of three GWAS pooled data on depression as well as GWAS data related to MASLD and GWAS data on four liver enzymes were used in this study. Our findings indicated a strong causal relationship between depression and MASLD (OR, 1.557; 95% CI, 1.097–2.211; *P* = 0.016). And we found a mediating effect of gamma-glutamyl transferase (GGT), alanine aminotransferase (ALT) and aspartate aminotransferase (AST). ALT 10% (95% CI: 7% - 13%, *P*< 0.0002). AST, 4.14% (95% CI: 2.34% - 5.94%, *P* < 0.05). GGT 0.19% (95% CI: 0.15% - 0.22%, *P*< 0.000000002). However, we did not find a mediating effect of alkaline phosphatase (ALP). Our inverse MR analysis did not reveal any causal relationship between MASLD and depression.

**Conclusions:**

The MR analysis revealed a positive causal relationship between depression and MASLD, while no reverse causal relationship was identified. Liver enzymes may mediate the role between depression and MASLD.

## Introduction

Non-alcoholic fatty liver disease (NAFLD) is a clinical and pathological syndrome characterized by the excessive accumulation of fat within liver cells. It is primarily caused by non-alcoholic factors and other non-specific liver injury factors (1). In 2020, metabolic dysfunction-associated steatotic liver disease (MASLD) was deemed a more appropriate term than NAFLD, as it better delineates the pathophysiology of this hepatic condition and its associated metabolic aberrations (2). This alteration extends beyond mere nomenclature, as it influences clinicians’ diagnostic approach to the disease. Moreover, the term “non-alcoholic” may lead to patient misconceptions regarding etiology, thereby hindering therapeutic rapport (3). Therefore, this manuscript adopts the term “ MASLD “. With the global prevalence of obesity and its associated metabolic syndrome on the rise (4), the incidence of MASLD is steadily increasing year by year. Epidemiological surveys have revealed that the prevalence of MASLD is approximately 25%. It is noteworthy that MASLD carries the potential to progress into high-risk conditions, including liver cirrhosis and hepatocellular carcinoma (5–7), Moreover, the progression of MASLD can ultimately lead to patient mortality. A recent study conducted in the United States has revealed a correlation between the growing prevalence of MASLD and the increasing mortality rate associated with liver disease (8). MASLD is gradually emerging as a global public health challenge (9). Given the intricate and unclear pathophysiology of MASLD, the primary emphasis in clinical management strategies revolves around prevention and lifestyle management. Extensive clinical studies have highlighted specific risk factors, including alcohol consumption, obesity, and infections caused by hepatitis B and C viruses, which may significantly contribute to the development of MASLD (10). Nevertheless, the association of most risk factors with the onset of MASLD remains uncertain and requires additional evidence for validation. Therefore, it is essential to shift our attention towards exploring other potential modifiable risk factors, including mental health (11). Patients with various mental disorders have a higher prevalence of MASLD, which often goes unnoticed as a significant contributor to reduced life expectancy and diminished quality of life (12).

Depression is a widespread psychological disorder that has a profound impact on an individual’s social, psychological functioning, and overall quality of life (13). According to a prevalence survey, the global number of individuals experiencing depression has surged by 49.86%, rising from 172 million in 1990 to 258 million in 2017 (14). Additionally, in certain regions, the lifetime prevalence of depression can reach as high as 21% (15). Depression serves as a significant risk factor for numerous diseases, including cardiovascular disease, dementia, migraines, and various others (16–18). The role of depression in the onset and progression of MASLD is gradually gaining attention as a research hotspot (19). Patients who experience both MASLD and depression typically have a more unfavorable prognosis (20, 21). Observational studies have provided substantial evidence of a significant correlation between the presence and severity of MASLD and depression (22, 23). However, it is important to note that there are some studies that contradict the findings reported in previous literature (24). Indeed, the relationship between depression and MASLD has not been thoroughly examined due to potential biases, such as confounding factors or reverse causality. The causal role of depression in the development of MASLD remains unclear. Further validation and exploration are needed to determine whether depression can induce the occurrence of MASLD.

Mendelian randomization (MR) is a method that utilizes genetic variables as instrumental variables to explore causal relationships between risk factors and outcomes (25).. With the availability of large-scale GWAS data, numerous reliable genetic variants are now accessible for MR investigations. Consequently, several studies have employed MR techniques to explore causal relationships between various traits (26). The randomness of genetic variation enables MR analysis to establish genetic inference regarding the correlation between exposure variables and outcomes. This unique feature helps mitigate the impact of potential confounding factors and reverse causality bias (27). Therefore, we conducted a two-sample MR analysis to investigate the relationship between depression and MASLD.

## Materials and methods

### Study design

**Figure 1:**
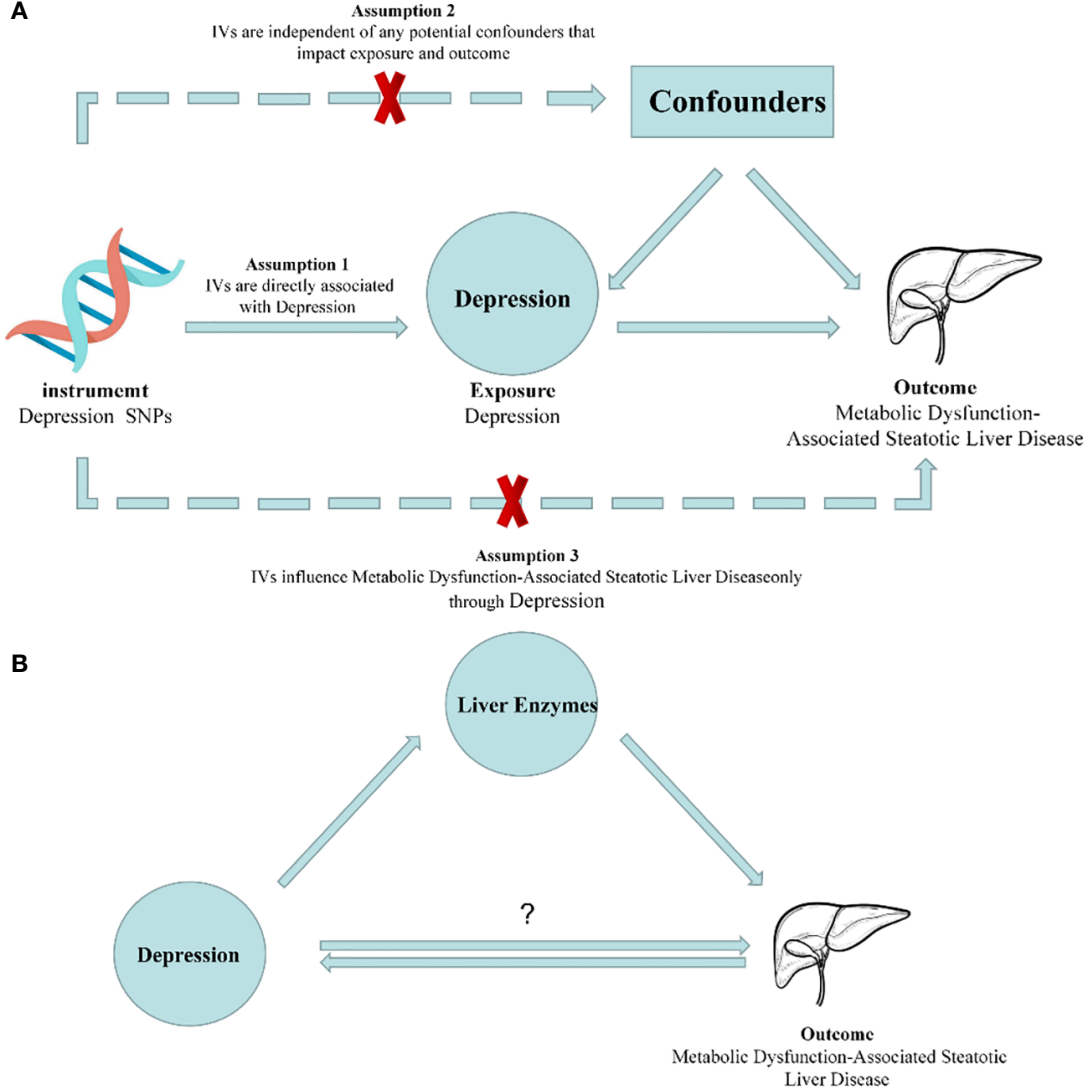
Diagrams illustrating associations examined in this study. **(A)** Rationale of Mendelian randomization study. IVs: Instrumental Variables. **(B)** Schematic for Mendelian randomization of mediating effects.

In this study, we employed a two-sample MR approach to assess the causal relationship between depression and MASLD, as well as liver enzyme levels. To ensure the utmost accuracy of our findings, it is of paramount importance to rigorously validate three key assumptions throughout the entire process (26). These three assumptions are as follows: Firstly, the instrumental variables (IVs) used, which are genetic variants, should not be associated with any confounding factors. Secondly, the genetic variants should not be related to any confounding factors. Thirdly, the genetic variants should exert their influence on the outcome risk solely through the exposure variable, rather than through alternative pathways. Since our study utilized summary-level data from publicly available genome-wide association studies (GWASs), it did not require approval from an ethics committee. However, it is important to note that the preliminary study, which provided the data for our work, obtained ethical approval and obtained appropriate consent from the patients (**Figure 1**).

### Data sources

For this study, we acquired the summary statistics data on depression from a recent genome-wide association meta-analysis conducted by Howard et al. This meta-analysis identified 102 independent single nucleotide polymorphisms (SNPs) that exhibited a significant association with depression. The study encompassed a large sample size of 807,553 individuals, comprising 246,363 cases and 561,190 controls, derived from three depression GWAS conducted on individuals of European ancestry (28). All of these SNPs reached genome-wide significance (*P* < 5*10-8). Subsequently, we selected appropriate proxy SNPs based on linkage disequilibrium (LD) levels with an *r* ^2^ < 0.001 and an LD distance > 10,000 kb and removed 24 SNPs with linkage disequilibrium. To ensure the fulfillment of assumptions 1 and 2 in the two-sample MR study design, we proceeded to merge the overlapping regions. This merging process confirmed that the instrumental variables (IVs) met the required criteria. However, it is important to note that 7 SNPs were lost during the merging process. Furthermore, we excluded 6 SNPs (rs10061069, rs12967143, rs1933802, rs2029865, rs2247523, and rs2876520) from the analysis due to their intermediate allele frequencies. Therefore, a total of 65 IVs were used in this two-sample MR study. The summary statistics data for the reverse MR analysis were also obtained from a genome-wide association meta-analysis conducted on individuals of European ancestry (29).. The study included a total of 322,580 individuals, including 113,769 cases and 208,811 controls.

The GWAS summary statistics data for MASLD were obtained from the FinnGen R8 release (30). The phenotype used in this study was “Nonalcoholic fatty liver disease”, and the GWAS included 342,499 Finnish adult participants, including 1908 cases and 340,591 controls. The eMERGE defined MASLD cases by Electronic health record (EHR) codes (ICD9: 571.5, ICD9: 571.8, ICD9: 571.9, ICD10: K75.81, ICD10: K76.0 and ICD10: K76.9). The FinnGen database defined MASLD using the International Classification of Diseases code K76.0. We obtained four liver enzymes, including alkaline phosphatase (ALP), alanine aminotransferase (ALT), aspartate aminotransferase (AST), and gamma-glutamyl transferase (GGT), which may be associated with MASLD (31). The summary data for liver enzymes and phenotype information can be obtained from the following link: https://docs.google.com/spreadsheets/d/1kvPoupSzsSFBNSztMzl04xMoSC3Kcx3CrjVf4yBmESU/edit?ts=5b5f17db#gid=227859291.

### Selection of instrumental variables

The strength of the IVs used in the MR analysis was assessed using the F statistic. This statistic measures the extent to which each SNP can explain the variance in the exposure variable. To ensure the inclusion of high-quality instruments, only SNPs with F statistics greater than 10 were included in the analysis (32). The following rigorous mathematical formula was adopted:


$$F=\left[\frac{R^{2}}{1-R^{2}}\right]*\left[\frac{n-k-1}{k}\right]$$

In MR analysis, K represents the number of SNPs used, N represents the sample size of the GWAS, and R2 represents the amount of exposure variance explained by each IV. If *F* is greater than 10, it shows that the instruments used in the study are strong enough. This means that the independent variables have a significant estimated effect, and the subsequent MR analysis won’t be affected by weak instrument bias. The IVs we selected all met the criterion of *F* > 10.

### Statistical analysis

In this study, we employed five methods for MR analysis, namely inverse variance weighted (IVW), MR-Egger, weighted median, weighted mode, and simple mode. These methods were utilized to comprehensively evaluate the potential relationships. However, IVW was selected as the primary analysis method for this study (33). The IVW method is utilized to examine causal relationships by conducting a meta-analysis of the Wald ratios associated with each included SNP. This approach can offer a reliable estimate of causal effects, even when a considerable portion, up to 50%, of the information in the analysis is derived from potentially invalid instrumental variables (34). Compared to the IVW method, the MR-Egger method generally yields larger standard errors for causal estimates and lower magnitudes of causal effects (35). This phenomenon is particularly noticeable when the correlation coefficient between SNPs and the exposure variable is similar or when the number of instrumental variables is limited. In such cases, the MR-Egger method tends to exhibit larger standard errors and lower causal estimates. Alternatively, the weighted median estimator can offer an unbiased test, especially in situations where up to 50% of the SNPs are considered invalid instruments (34). Regarding the weighted mode method, its reliability is contingent upon the condition that the largest subset of instruments with similar causal effects remains valid.

When performing a MR analysis, it is crucial to evaluate the robustness of the results, the reliability of the conclusions, and the potential for bias in the analysis. To ensure the validity of MR assumptions, it is common practice to conduct multiple sensitivity analyses to assess heterogeneity and pleiotropy within the genetic instruments. In this study, we employed the Cochrane Q test to evaluate heterogeneity among the instrumental variables. A p-value of less than 0.05 was considered indicative of the presence of heterogeneity (36). In addition, we employed the MR-PRESSO and MR-Egger intercept methods to detect the presence of horizontal pleiotropy among the SNPs (37). If the results of the “leave-one-out” analysis are inconsistent with the causal effect analysis, it indicates that there might be an influence on the estimated causal effect (38). Finally, we utilized scatter plots and forest plots to visually present the results of the MR analysis.

We did all the analyses on the R software (version 4.2.1.; R Foundation for Statistical Computing, 2022) and RStudio(version, 2022.07.2 + 576), and R package TwoSampleMR and MR-PRESSO were used.

### Mediation effects of liver enzymes

Furthermore, we will multiply the estimated effects of depression on each hepatic enzyme by the estimated effects of each hepatic enzyme on MASLD to obtain the individual mediating effects of each hepatic enzyme. Subsequently, we will divide the mediating effects by the total effect of depression on MASLD to determine the proportions mediated by each mediating factor. Lastly, we will employ the coefficient product method to calculate the standard error and assess the statistical significance.

## Results

**Figure 2:**
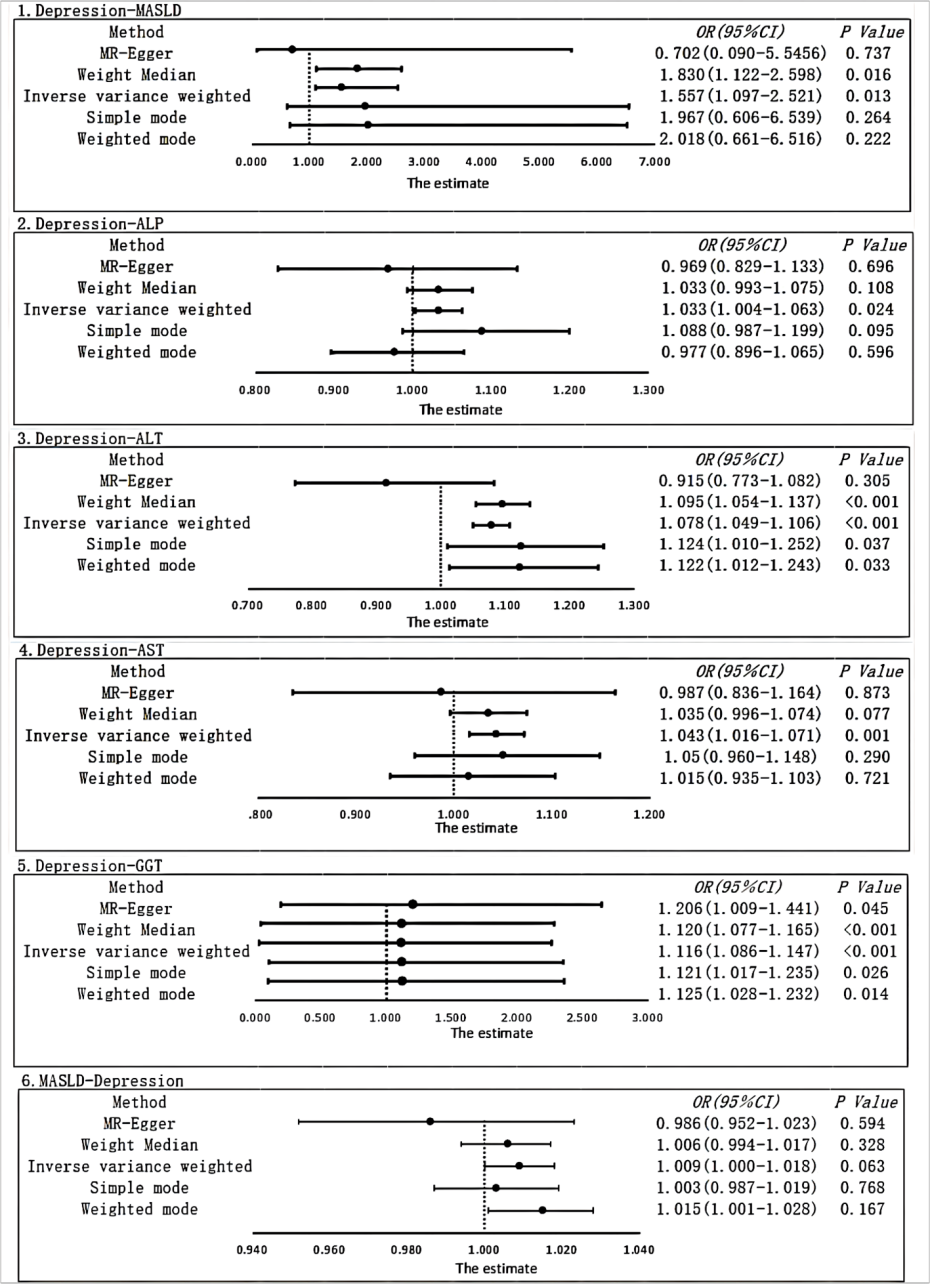
Using different MR methods to estimate the causal relationship between depression and metabolic dysfunction-associated steatotic liver disease. The forest plot shows the causal effect of each single SNP.

We employed strict screening criteria (*P* < 5*10-8, *r*2 < 0.001, kb = 10000) to identify genetic variants, resulting in a total of 65 SNPs used as IVs for depression in the current investigation. In the end, we analyzed the results by two-sample MR analysis, in which IVW analysis (OR, 1.557; 95% CI, 1.097–2.211; *P* = 0.016) showed that there was a strong causal relationship between depression and MASLD. The results obtained from the weighted median analysis (OR, 1.830; 95% CI, 1.122–2.984; *P* = 0.016) were found to be similar to the results obtained from the IVW method. However, the results from the weighted mode (OR, 2.018; 95% CI, 0.661–6.159; *P* = 0.222), MR-Egger (OR, 0.702; 95% CI, 0.090–5.456; *P* = 0.737), and simple mode (OR, 1.967; 95% CI, 0.606–6.385; *P* = 0.264) analyses showed no significant association (**Figure 2**).

In the sensitivity analysis, we obtained no evidence of directional pleiotropy among the IVs (*p* = 0.442) from the MR-Egger regression analysis. Moreover, there was no significant difference between the results of the Cochrane’s Q test, IVW, and MR-Egger analyses (*p* > 0.05) (as shown in **Table 1**). Furthermore, the symmetry of the funnel plot did not indicate any evidence of publication bias. Additionally, the leave-one-out analysis showed that there was no bias from individual SNPs.

**Table 1:** Results of heterogeneity and sensitivity test.

Exposure	Outcome	Methods	*P* of Pleiotropy	*P* of Cochrane Q
Depression	MASLD	MR Egger IVW	0.442	0.346 0.359
Depression	ALP	MR Egger IVW	0.417	0.340 0.352
Depression	ALT	MR Egger IVW	0.059	0.382 0.286
Depression	AST	MR Egger IVW	0.504	0.933 0.939
Depression	GGT	MR Egger IVW	0.442	0.401 0.410
MASLD	Depression	MR Egger IVW	0.425	0.923 0.445

MR, Mendelian randomization; MASLD, Metabolic dysfunction-associated steatotic liver disease; IVW, inverse-variance weighted; ALP, alkaline phosphatase; ALT, alanine aminotransferase; AST, aspartate aminotransferase; GGT, gamma-glutamyl transferase.

**Figure 3:**
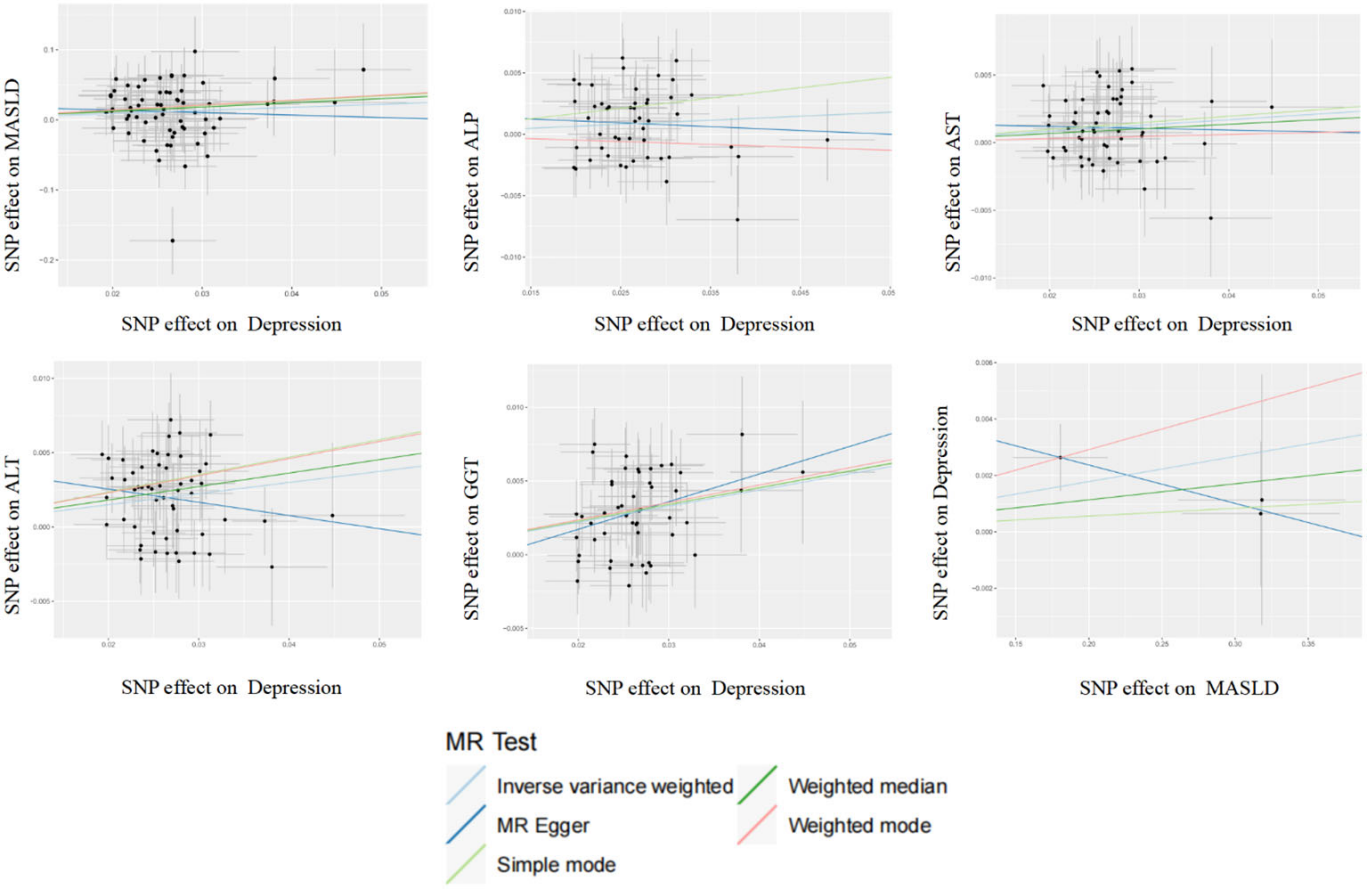
Scatter plot analysis of the genetic correlation between depression and metabolic dysfunction-associated steatotic liver disease using different MR analysis methods.

**Figure 4:**
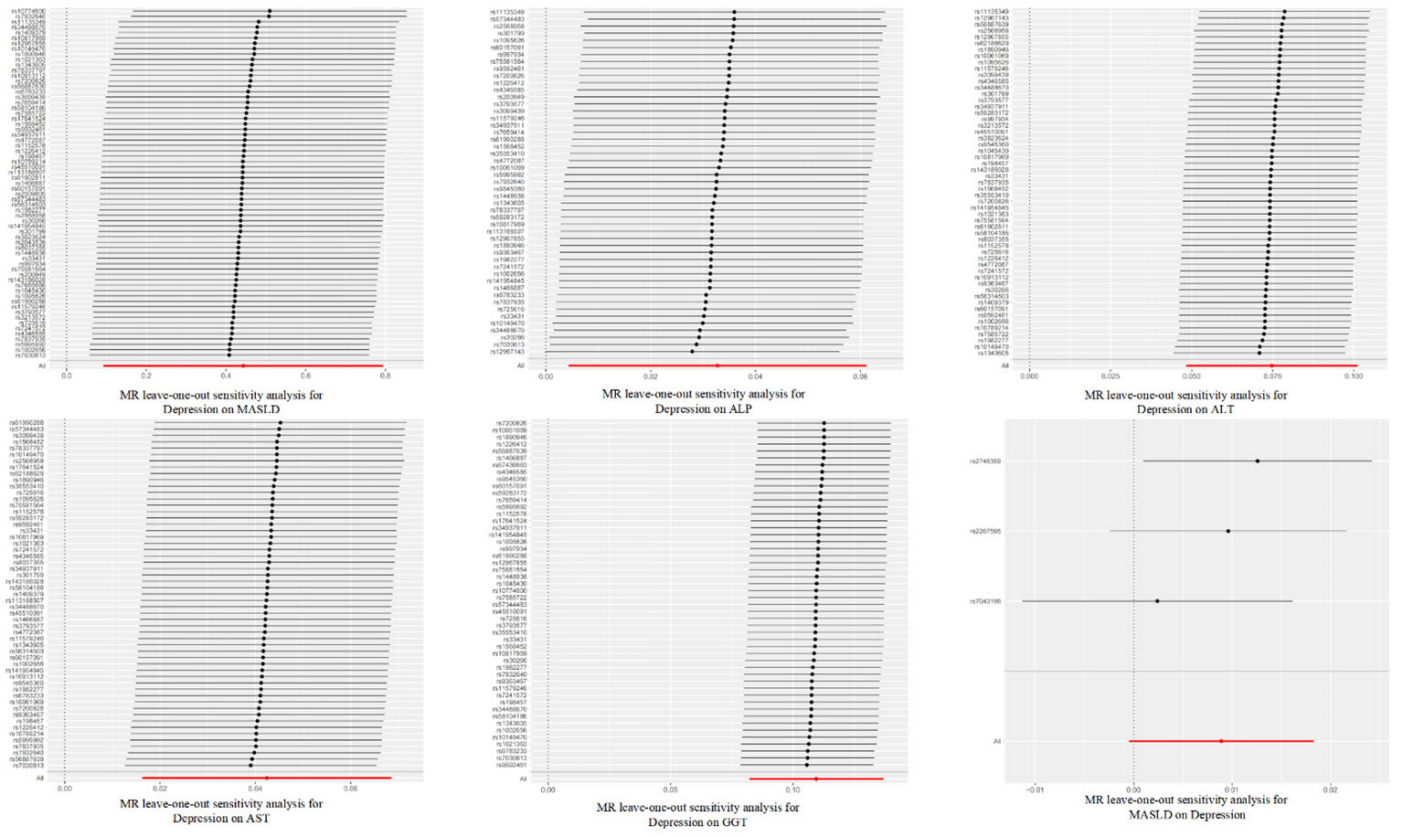
Leave-one-out sensitivity analysis for the relationship between depression and metabolic dysfunction-associated steatotic liver disease. The red line indicates the estimate of the IVW test.

**Figure 5:**
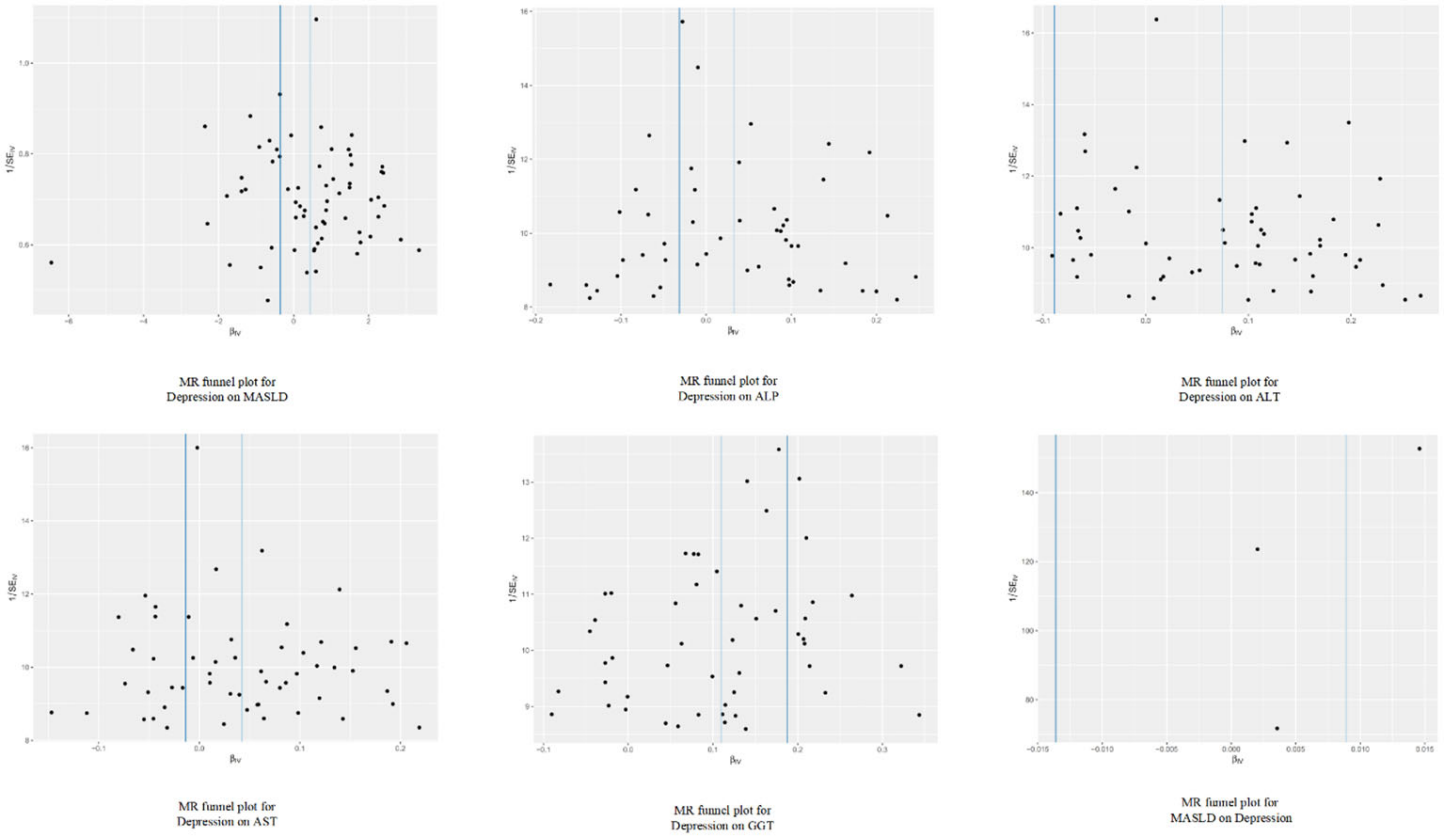
Funnel plot analysis of the genetic correlation relationship between depression and metabolic dysfunction-associated steatotic liver disease.

After correcting for pleiotropy and heterogeneity using Radial MR and MR-PRESSO, the MR analysis revealed a significant positive correlation between depression and increased risk of liver damage. All four liver enzymes that we included demonstrated a causal relationship in the primary MR analysis (IVW). The results of the MR analysis showed that the odds ratios (ORs) for the four liver enzymes were as follows: ALP (OR, 1.033; 95% CI, 1.004–1.063; *P* = 0.024), ALT (OR, 1.078; 95% CI, 1.049–1.106; *P* < 0.001), AST (OR, 1.043; 95% CI, 1.016–1.071; *P* = 0.001), and GGT (OR, 1.116; 95% CI, 1.086–1.147; *P* < 0.001). Sensitivity analyses did not reveal any significant differences. Regarding the reverse MR results, the IVW analysis did not yield statistically significant results (OR, 1.009; 95% CI, 1.000–1.018; *P* = 0.063). The above results are all presented through visual graphics (**Figures 3**–**5**).

### Mediation effects of liver enzymes

Our results indicate that GGT plays a crucial mediating role, while ALT and AST further contribute to the modulation of depression on MASLD. The mediating proportion for ALT was estimated at 10% (95% CI: 7% - 13%, *P* < 0.0002). For AST, the mediating proportion was estimated at 4.14% (95% CI: 2.34% - 5.94%, *P* < 0.05). GGT exhibited a substantial mediating effect, with a proportion of 0.19% (95% CI:0.15% - 0.22%, *P* < 0.000000002), accounting for 17.1% of the total mediation. However, we did not find a mediating effect of ALP. The mediating proportion for ALP was estimated at 2.05% (95% CI: -7.48% - 11.57%, *P* =0.673) (**Table 2**).

**Table 2:** Results of mediation effects of liver enzymes.

Mediators	Beta(95%CI)	*P-value*
ALT	10% (7% - 13%)	<0.001
AST	4.14%(2.34%-5.94%)	< 0.05
GGT	0.19%(0.15% - 0.22%)	<0.001
ALP	2.05%(-7.48%-11.57%)	0.673

## Discussion

We utilized a two-sample MR approach to thoroughly investigate the causal impact of depression on the incidence of MASLD. Our analysis revealed a statistically significant causal relationship between genetic susceptibility to depression and the risk of MASLD. Sensitivity analysis results further confirmed the reliability and robustness of our findings. Additionally, we conducted a reverse MR analysis, which indicated no statistically significant causal relationship between genetic susceptibility to MASLD and the risk of depression. Our findings also indicate that ALT, AST, and GGT play significant causal moderating roles in the relationship between depression and MASLD.

The relationship between depression and MASLD is currently not well understood (22–24). Observational studies have yielded inconsistent findings regarding the relationship between depression and MASLD. While some cohorts suggest that depression may be a risk factor for the progression of MASLD, there is no clear consensus in the current literature (39). However, in other cohorts, there is no observed relationship between the incidence of depression and the progression of MASLD (24, 40). Additionally, a cohort study by Labenz C et al. (41) demonstrated that MASLD increases the risk of anxiety and depression. In a cross-sectional study conducted by Youssef N.A. et al. (42), it was found that 53% of MASLD patients had subclinical depression, while 14% had clinical depression. Furthermore, Li, H. et al. (43) found in a cohort study that patients with abnormal mental status had a high prevalence of liver steatosis and fibrosis. When compared to MASLD patients without depression, those with comorbid depression and MASLD exhibited more severe hepatic steatosis on histology and poorer treatment outcomes (21). These recent studies indicate a bidirectional relationship between depression and MASLD.

Early epidemiological studies on the relationship between depression and MASLD were predominantly case-control or cross-sectional in design, lacking a clear temporal sequence and making it challenging to establish a causal relationship. Additionally, previous observational studies were limited by small sample sizes, difficulties in avoiding reverse causality, and the presence of confounding factors. Furthermore, certain studies have indicated that individuals taking antipsychotic medications have a higher incidence of MASLD compared to the general population (44). However, unconventional antipsychotic medications are prone to inducing hepatic injury and metabolic disturbances, among other adverse effects (45). This makes it difficult to ascertain whether it is depression itself or the medication that is influencing metabolic function. The presence of confounding factors in observational studies has further complicated the issue, making it challenging to determine the true relationship between depression and MASLD. However, with advancements in research designs, the use of bidirectional MR analysis methods has emerged as a promising approach to better elucidate the causal relationship between exposure and outcome in current studies.

In our study, we exclusively uncovered a causal relationship between depression and MASLD, ruling out any reverse causality. Furthermore, the causal association between depression and MASLD is likely mediated by hepatic enzymes. Notably, GGT demonstrates the highest proportion of mediation, aligning with the findings from the clinical observations. In a clinical observational report by Karen, K et al. (46), it was indicated that an elevation in negative emotional scores is contingent upon higher levels of GGT. Although liver enzymes are correlated with inflammation, they are not linked to the metabolic dysregulation typically found in MASLD patients. Nevertheless, in up to 20–25% of MASLD cases, hepatic inflammation emerges, denoted as metabolic associated steatohepatitis (MASH) (47). This may lead to further hepatic complications, such as advanced fibrosis, cirrhosis, ultimately culminating in hepatocellular carcinoma (HCC) (48). Depression patients commonly undergo systemic inflammation phases. Liver damage may occur at this stage (49). An observational study revealed that among 382 patients with depression, the incidence of metabolic syndrome was 27.7%, with liver enzymes markedly elevated compared to patients with normal indices (50). And some studies have shown that improving liver damage can improve depressive states. Yan et al. (51)discovered that Nootkatone could ameliorate anxiety and depressive-like behaviors induced by liver damage in mice. Jung et al. (52)discovered through lipidomics that acupuncture treatment in the chronic restraint stress (CRS) induced depression mouse model effectively alleviates depressive-like behavior, reduces hepatic enzymes, and ameliorates hepatic lipid metabolism. However, it is important to acknowledge that the absence of support for a reverse causal relationship in our study could be attributed to limitations in the study design or variations in the population data. It is possible that differences in population data, such as variances in race, may have contributed to the observed disparities. In the European population, there may be observed correlations as well as genetic correlations between MASLD and depression, but no evidence of a reverse causal relationship. This relationship can be explained through various mechanisms, and one possible explanation is the role of inflammatory factors. Both depression and inflammation can mutually exacerbate each other (53). In a recent study conducted by Chan, K. L. et al., it was found that both central and peripheral inflammation were associated with metabolic syndrome and severe depression. This suggests that inflammation plays a significant role in the development of both MASLD and depression (54, 55). Based on the information presented, it is reasonable to speculate that depression may impact MASLD through inflammatory factors. Additionally, there are shared risk factors between MASLD and depression, such as diet and exercise, which play significant roles in the development, progression, and treatment of depression. Similarly, these lifestyle-related risk factors also influence the incidence and progression of MASLD (56). The relationship between depression and MASLD may be mediated by insulin resistance. Depression may lead to immune-mediated destruction of pancreatic beta cells, resulting in insulin resistance and diabetes, both of which are important risk factors for MASLD (9, 57, 58)

Depression and MASLD frequently coexist and typically require long-term treatment. Gender and age have been identified as potential factors that may contribute to this relationship. Research studies have indicated that postmenopausal women may be more susceptible to experiencing both depression and MASLD (59–61). Women with depression, particularly those aged 50 and above, may require heightened clinical vigilance to prevent the development of MASLD. The findings of our study indicate a potential causal relationship between depression and the risk of MASLD. These findings underscore the importance for healthcare providers to closely monitor the development of MASLD in patients with depression.

Our MR study offers several advantages. Firstly, we employed the MR analysis method, which utilizes instrumental variables, specifically high correlation strength SNPs with an F-statistic greater than 10. This approach strengthens the validity of our findings by mimicking the design of a randomized controlled trial. Our study is based on a large-scale GWAS to assess potential causal effects, ensuring the reliability of the sample size. Additionally, we utilized data exclusively from GWAS databases, specifically from European population samples, which effectively minimizes bias stemming from population heterogeneity. These measures enhance the internal validity of our findings. Furthermore, our analytical results carry significant implications for healthcare policy. By uncovering the causal relationship between depression and MASLD, our study may influence public health policies focused on prevention and treatment.

However, there are several parts of this experiment that need to be refined or studied in depth. Firstly, we found that the proportion of women with depression and MASLD was higher than that of men. However, since our data came from public databases, we were unable to conduct subgroup analyses for specific factors such as age and gender. Secondly, it is important to recognize that the relationship between depression and MASLD is influenced by multiple factors, extending beyond genetic determinants alone. While our study made efforts to control for confounding variables, we acknowledge that there may still be residual interference from factors that could impact our findings, including psychological and social factors such as occupation and family dynamics. Depression and MASLD are chronic and progressive conditions, and their development from mild to severe stages can be influenced by numerous external confounding factors. These factors may potentially impact the outcomes of our study. Thirdly, it is important to note that the GWAS data we utilized consisted solely of participants of European descent. Therefore, caution should be exercised when extrapolating our results to other populations, as further investigation is required to determine the generalizability of our findings.

## Conclusions

In summary, our study conducted bidirectional MR analysis in the European population, revealing a causal link between susceptibility to depression and an elevated risk of MASLD. However, the reverse MR analysis yielded results indicating no statistically significant causal relationship between genetic susceptibility to MASLD and the risk of depression.

## Data availability statement

Publicly available datasets were analyzed in this study. This data can be found here: IEU OpenGWAS project, https://gwas.mrcieu.ac.uk; FinnGen, https://www.finngen.fi/en; and NCBI, https://www.ncbi.nlm.nih.gov/pmc/articles/PMC6522363.

## Ethics statement

The Mendelian randomization analysis of this study was based on publicly available data and obtained ethical approval. The studies were conducted in accordance with the local legislation and institutional requirements. The participants provided their written informed consent to participate in this study.

## Author contributions

WL: Writing – original draft, Writing – review & editing, Conceptualization, Data curation, Methodology, Validation, Visualization. KZ: Conceptualization, Writing – review & editing. TL: Conceptualization, Writing – review & editing. YZ: Methodology, Visualization, Writing – review & editing. ZH: Validation, Visualization, Writing – review & editing. JQZ: Formal analysis, Investigation, Writing – review & editing. JH: Conceptualization, Methodology, Writing – review & editing. ZS: Conceptualization, Methodology, Writing – review & editing. JZ: Conceptualization, Resources, Supervision, Writing – review & editing. FD: Project administration, Resources, Supervision, Writing – review & editing.
